# Eugenol works synergistically with colistin against colistin-resistant *Pseudomonas aeruginosa* and *Klebsiella pneumoniae* isolates by enhancing membrane permeability

**DOI:** 10.1128/spectrum.03666-22

**Published:** 2023-09-14

**Authors:** Jingchun Kong, Yue Wang, Zhuocheng Yao, Yishuai Lin, Yi Zhang, Yijia Han, Tieli Zhou, Jianzhong Ye, Jianming Cao

**Affiliations:** 1 Department of Medical Lab Science, School of Laboratory Medicine and Life Science, Wenzhou Medical University, Wenzhou, Zhejiang Province, China; 2 Department of Clinical Laboratory, Key Laboratory of Clinical Laboratory Diagnosis and Translational Research of Zhejiang Province, The First Affiliated Hospital of Wenzhou Medical University, Wenzhou, Zhejiang Province, China; University of Exeter, Exeter, United Kingdom

**Keywords:** synergy, eugenol, colistin-resistant, *Pseudomonas aeruginosa*, *Klebsiella pneumoniae*, biofilm

## Abstract

Colistin is a potent antibiotic for the treatment of carbapenem-resistant Gram-negative bacteria and is considered a last-resort drug. Unfortunately, the incidence of colistin-resistant bacteria isolated from patients is continuously growing due to clinical reuse of colistin. In this study, we found that the combination of colistin and eugenol has a significant synergistic antibacterial effect and reverses the sensitivity of colistin-resistant *Pseudomonas aeruginosa* and *Klebsiella pneumoniae* against colistin, as confirmed by checkerboard and time-kill assays. Crystal violet staining and scanning electron microscopy revealed colistin and eugenol’s synergistic antibiofilm action. Concerning the synergy mechanism, the results revealed that the combination of eugenol and colistin increases membrane permeability and causes considerable membrane damage, further inhibiting bacteria synergistically. Meanwhile, up to 500 µg/mL of eugenol is non-toxic to RAW 264.7 cells, and the colistin/eugenol combination is also efficacious *in vivo*, as demonstrated by the *Galleria mellonella* infection model. Our findings indicate that the colistin/eugenol combination is a viable treatment option for colistin-resistant *P. aeruginosa* and *K. pneumoniae* clinical infections.

**IMPORTANCE:**

Colistin is used as a last resort for severe infections caused by multidrug-resistant Gram-negative bacteria, however, colistin resistance is increasing. As a result, we investigated the synergistic effect of eugenol/colistin combination, and the results revealed significant antibacterial and antibiofilm action. Eugenol may help clinical colistin-resistant *Pseudomonas aeruginosa* and *Klebsiella pneumoniae* recover their susceptibility. These findings suggest that combining eugenol and colistin may be a viable treatment option for colistin-resistant pathogen clinical infections.

## INTRODUCTION

Antibiotic resistance has become increasingly severe in recent years as a result of antibiotic abuse or misuse, resulting in millions of infections and tens of thousands of deaths (1, 2). Multidrug-resistant (MDR), extensively drug-resistant (XDR), and pandrug-resistant (PDR) bacteria further enhanced the danger level due to the horizontal transfer of antibiotic resistance genes (3). WHO has listed *Enterococcus* spp., *Staphylococcus aureus*, *K. pneumoniae*, *Acinetobacter baumannii*, *P. aeruginosa*, and *Enterobacter* spp. (ESKAPE) as the most tendency of antibiotic resistance, and these pathogens are the most commonly detected microbes in clinical settings (4). In addition, mobile resistance components could be transferred across these pathogens or to other species, which exacerbates the revolution of antibiotic resistance (5).

Biofilm is the major outcome of the bacteria quorum sensing (QS) system, which shields and prevents bacteria from antibiotic pressure (6). Strong biofilm producers or high virulence strains are more competent to colony in the invasion of sites and cause more obstinate infections (7). Therefore, anti-biofilm production is a feasible target for anti-infection.

Colistin (Polymyxin E), a cationic lipopeptide, was shelved in the past owing to the nephrotoxicity and neurotoxicity (8). However, colistin was reapplied to cope with Gram-negative bacteria that were resistant to carbapenem (9). Unfortunately, the isolation rate of colistin-resistant strains is increasing, which is caused by several resistance mechanisms (9). If this situation is not successfully managed, humanity will soon reach the post-antibiotic era (10, 11). Hence, novel alternative approaches to combat superbugs, including antimicrobial peptides, antimicrobial nanomaterials, bacteriophages, and phytochemicals (12), are continually being developed and utilized.

Eugenol (4-Allyl-2-methoxyphenol), an aromatic phenolic compound, is the main active ingredient in natural essential oils (EOs) (13). Eugenol possesses several pharmacological effects, including anti-inflammatory, antioxidant, anti-parasitic, and antibacterial activities (14, 15). The -OH group of its molecule confers antibacterial action on eugenol (16). Synergistic effects were also reported in its combination with fluconazole, azithromycin, cefotaxime, ciprofloxacin, and vancomycin (17 – 19). In a prior study, eugenol combined with colistin showed synergistic antibacterial effect against *Escherichia coli* harboring *mcr-1* (20). The *mcr-1* gene located on the plasmid encodes Phosphoethanolamine (PEtN) transferase, which modifies lipid A by adding PEtN to reduce the negative charge of the membrane, thereby mediating colistin resistance (21). These reports demonstrate that eugenol can be utilized as an antibiotic adjuvant. It is still uncertain whether eugenol can improve the efficacy of colistin against *P. aeruginosa* and *K. pneumoniae*.

In this study, we evaluated the *in vitro* and *in vivo* antibacterial and anti-biofilm activities of eugenol/colistin combination against clinical isolates of *P. aeruginosa* and *K. pneumoniae*. Furthermore, we identified the synergistic mechanism between these two drugs, aiming to provide novel treatment options for colistin-resistant infections.

## RESULTS

### MDR phenotype of *P. aeruginosa* and *K. pneumoniae*

The antibiotic susceptibility of the 14 tested isolates was shown in Table 1; ATCC 25922 and ATCC 27853 served as controls for *P. aeruginosa* and *K. pneumoniae*, respectively. Twelve of the 14 isolates displayed MDR phenotype for β-lactams, quinolones, and aminoglycosides. All isolates showed different resistance levels to colistin. The minimum inhibitory concentrations (MICs) of eugenol for all *K. pneumoniae* isolates were 1,000 µg/mL, while the MICs for *P. aeruginosa* strains were all above 1,000 µg/mL.

**TABLE 1:** The MICs (μg/mL) value of colistin-resistant clinical isolates^*a*^ ^,^ ^*b*^

Species	Isolates	Antibiotics										
		ATM	CAZ	FEP	IPM	CIP	LVX	GEN	TOB	CAZ/AVI	COL	EG
		Breakpoints (S-R μg/mL)										
		8–32	8–32	8–32	2–8	0.5–2	1–4	4–16	4–16	8/4–16/4	2–4	
*P. aeruginosa*	ATCC27853	4	1	2	1	0.25	0.5	1	1	1/4	1	
	TL1671	8	4	8	2	0.25	1	2	1	1/4	16	>1,000
	**TL1736**	4	4	2	16	1	1	32	8	2/4	32	>1,000
	**TL1744**	32	32	16	16	32	8	>128	32	1/4	16	>1,000
	TL2314	16	32	16	4	0.5	2	8	2	4/4	16	>1,000
	**TL2917**	32	16	16	16	0.25	2	8	8	8/4	16	>1,000
	**TL2967**	128	16	32	16	8	16	8	8	16/4	4	>1,000
	**TL3008**	4	2	4	16	0.5	1	16	4	2/4	128	>1,000
	**TL3086**	128	16	16	>128	16	8	>128	128	2/4	256	>1,000
*K. pneumoniae*		4–16	4–16	2–16	1–4	0.25–1	0.5–2	4–16	4–16	8/4–16/4	2–4	
	ATCC25922	0.06	0.12	0.016	0.06	0.008	0.008	0.5	1	0.06/4	1	
	**FK169**	1	16	0.5	4	2	1	1	64	0.25/4	>128	1,000
	**FK1342**	128	>128	>128	0.25	1	0.5	1	4	0.12/4	128	1,000
	**FK3994**	>128	128	>128	32	>128	64	>128	>128	2/4	64	1,000
	**FK6556**	64	64	64	16	4	8	16	16	1/4	8	1,000
	**FK6663**	>128	>128	>128	32	>128	>128	>128	>128	0.5/4	8	1,000
	**FK6696**	>128	64	>128	128	>128	64	>128	>128	4/4	64	1,000

^*a*^ MIC, minimum inhibitory concentration; ATM, aztreonam; CAZ, ceftazidime; FEP, cefepime; IPM, imipenem; CIP, ciprofloxacin; LVX, levofloxacin; GEN, gentamicin; TOB, tobramycin; CAZ/AVI ceftazidime/avibactam; COL, colistin, EG, eugenol.

^*b*^ Isolates in bold represent MDR phenotype.

### The synergistic effect was analyzed by checkerboard assay

The synergistic effects of colistin and eugenol against clinical isolates were shown in Table 2. The fractional inhibitory concentration indexs (FICIs) for these strains ranged from 0.078 to 0.3125, demonstrating that the combination is effective against colistin-resistant *P. aeruginosa* and *K. pneumoniae*. The MIC of colistin in combination reduced 4–512 folds, and the majority of the isolates (12/14) regained colistin susceptibility in the presence of eugenol.

**TABLE 2:** The MICs and FICIs value for colistin/eugenol combination against colistin-resistant *P. aeruginosa* and *K. pneumoniae* clinical isolates^*a*^

Isolates	MIC (μg/mL)		FIC (μg/mL)		FICI	Interaction
	Colistin	Eugenol	Colistin	Eugenol		
TL1671	16	>1,000	0.5	125	<0.156	Synergy
TL1736	32	>1,000	2	31.25	<0.09	Synergy
TL1744	16	>1,000	0.25	62.5	<0.078	Synergy
TL2314	16	>1,000	1	125	<0.187	Synergy
TL2917	16	>1,000	0.5	125	<0.156	Synergy
TL2967	4	>1,000	1	31.25	<0.281	Synergy
TL3008	128	>1,000	2	125	<0.140	Synergy
TL3086	256	>1,000	8	125	<0.156	Synergy
FK169	>128	1,000	0.5	250	<0.254	Synergy
FK1342	128	1,000	0.25	250	0.2519	Synergy
FK3994	64	1,000	2	125	0.156	Synergy
FK6556	8	1,000	2	62.5	0.3125	Synergy
FK6663	8	1,000	0.5	250	0.3125	Synergy
FK6696	64	1,000	4	250	0.3125	Synergy

^*a*^ FIC, fractional inhibitory concentration; FICI, fractional inhibitory concentration index.

### Time-kill assays

**Fig 1:**
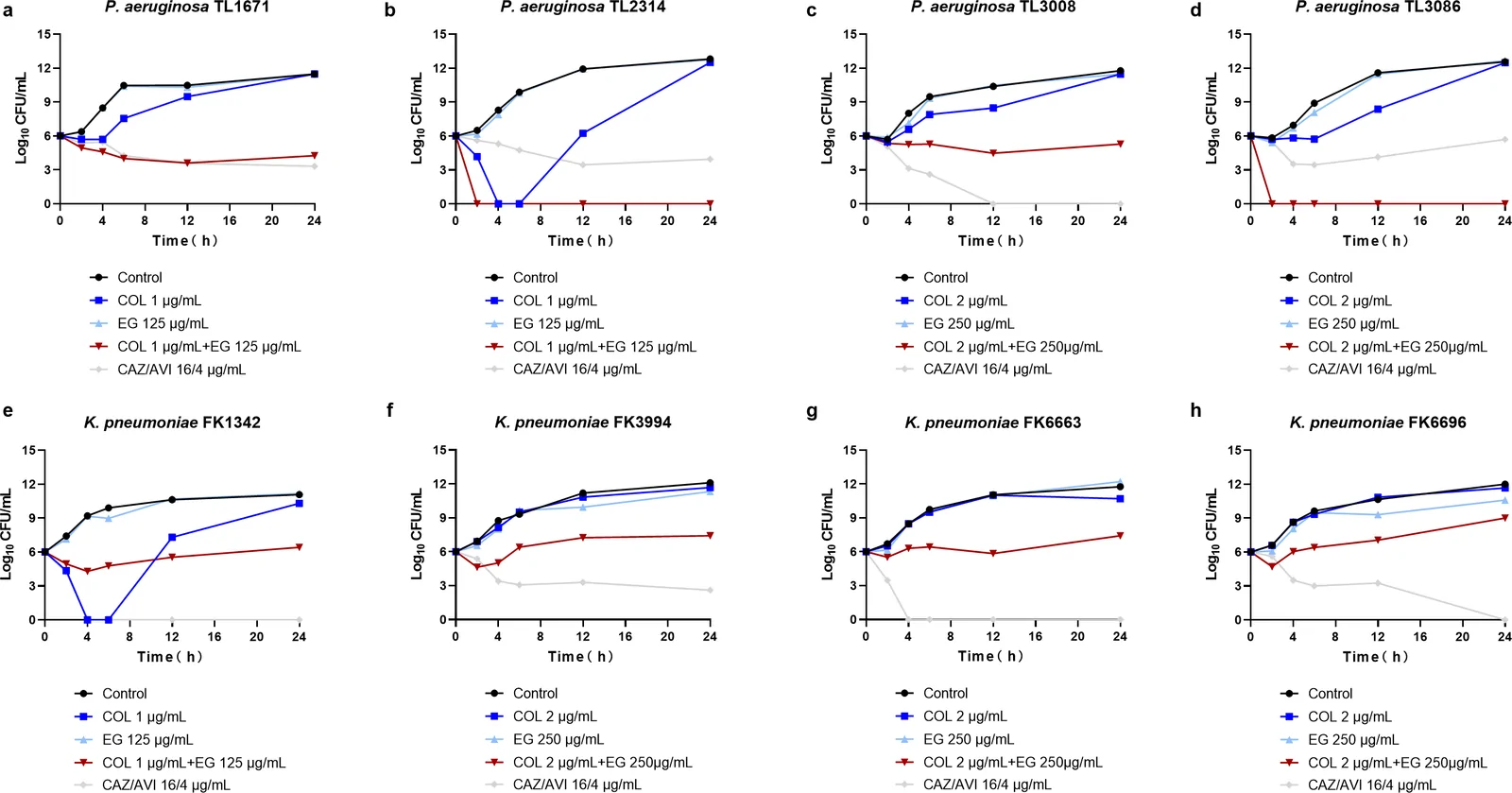
Time-kill curves for four treatments against colistin-resistant *P. aeruginosa* and *K. pneumoniae*. (**a–d**) log_10_ CFU/mL of four colistin-resistant *P. aeruginosa* isolates with various treatments for 24 h. (**e–h**) log_10_ CFU/mL of four colistin-resistant *K. pneumoniae* isolates with various treatments for 24 h. COL, colistin; EG, eugenol.

Time-kill assays were performed to further investigate the synergy of colistin and eugenol against colistin-resistant bacteria. The concentrations of these two drugs were determined using a checkerboard assay with FICI ≤ 0.5. As shown in Fig. 1, colistin treatments alone modestly inhibited the growth of partial isolates within 12 h, but had no effect on bacterial growth after 24 h. Eugenol alone had minimal inhibitory impact on any isolates. However, as compared to other treatments, the combined use of the two drugs has a significant antibacterial effect. According to the results depicted in the graph, the combined treatment was able to completely kill *P. aeruginosa* TL2314 and TL3086 within 2 h. For the other two strains of *P. aeruginosa* and four strains of *K. pneumoniae*, the combined treatment initially resulted in a modest reduction in bacterial numbers, followed by regrowth of the bacteria. However, the combined treatment also significantly inhibited bacterial growth over time, indicating its potential efficacy as an antibacterial therapy.

### The synergistic effect on biofilm formation and eradication

**Fig 2:**
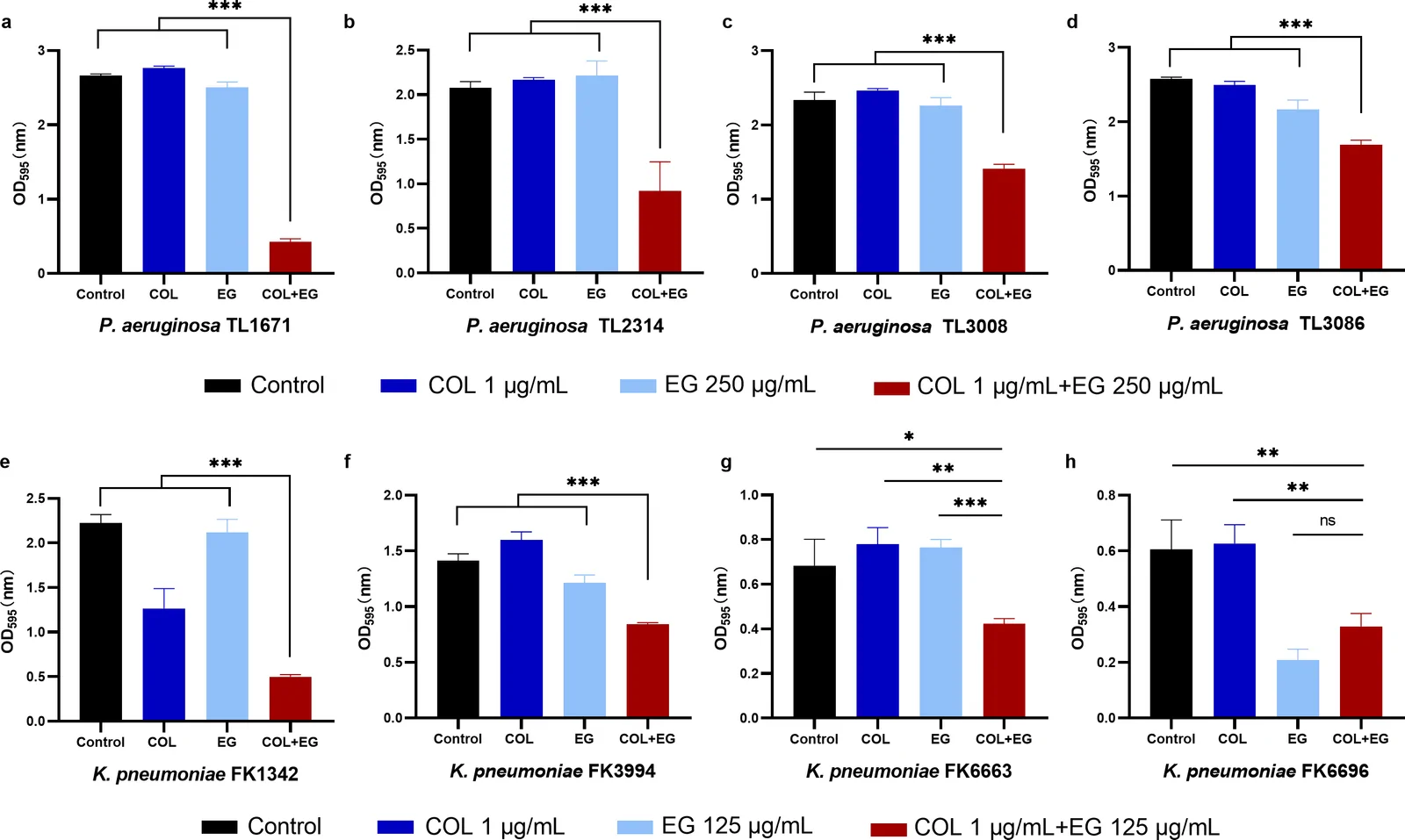
Eugenol, colistin, and their combination prevent *P. aeruginosa* and *K. pneumoniae* biofilm development. (**a–d**) No treatment, colistin 1 µg/mL, eugenol 250 µg/mL, and the combination were set for four *P*. *aeruginosa* isolates. (**e–h**) No treatment, colistin 1 µg/mL, eugenol 250 µg/mL, and the combination were set for four *K*. *pneumoniae* isolates. The drug concentration was determined using the checkerboard assay with FICI ≤ 0.5. *P* < 0.05 (*), *P* < 0.01 (**), and *P* < 0.001 (***) denote significance. COL, colistin; EG, eugenol; ns, nonsignificance.

Crystal violet staining was used to assess the effect of colistin/eugenol combination on biofilm development and eradication. As shown in Fig. 2, colistin/eugenol therapy significantly inhibited the biofilm of tested isolates when compared to the positive control and single-drug treatment (*P* < 0.05).

**Fig 3:**
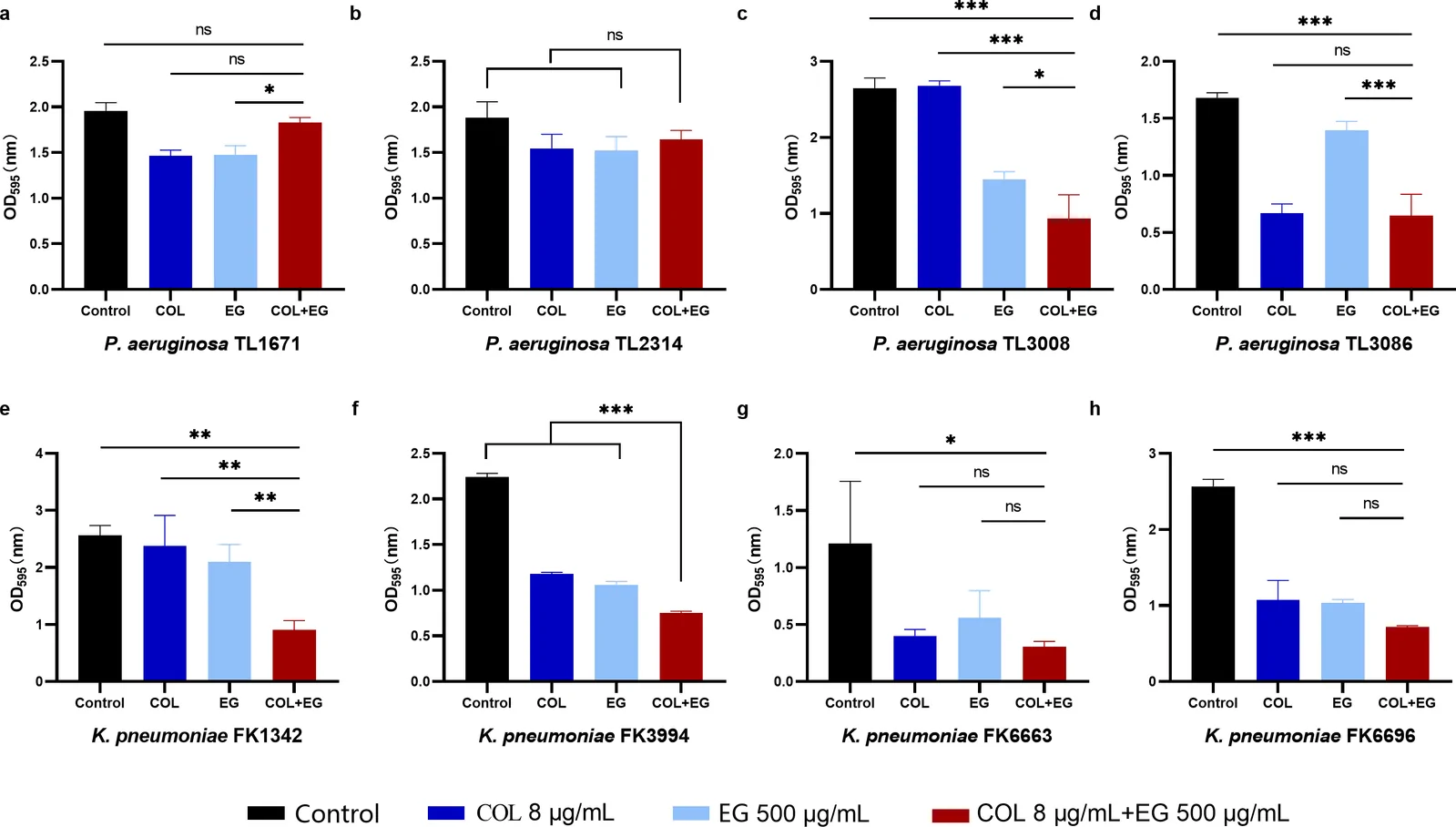
Eradication effect of eugenol combined with colistin on *P. aeruginosa* and *K. pneumoniae* mature biofilm. (**a–h**) No treatment, colistin 8 µg/mL, eugenol 500 µg/mL, and the combination were set for four *P*. *aeruginosa* and four *K*. *pneumoniae* isolates. The drug concentration was derived from the checkerboard assay with FICI ≤ 0.5. *P* < 0.05 (*), *P* < 0.01 (**), and *P* < 0.001 (***) denote significance. COL, colistin; EG, eugenol; ns, nonsignificance.

Following the investigation of biofilm formation, eradication of the colistin/eugenol combination on colonized biofilm was also studied. As shown in Fig. 3, as compared to the single treatment group, the combination group eliminated the produced biofilm on a subset of test isolates (3/8 isolates, *P*＜0.05).

### Scanning electron microscopy analyses

**Fig 4:**
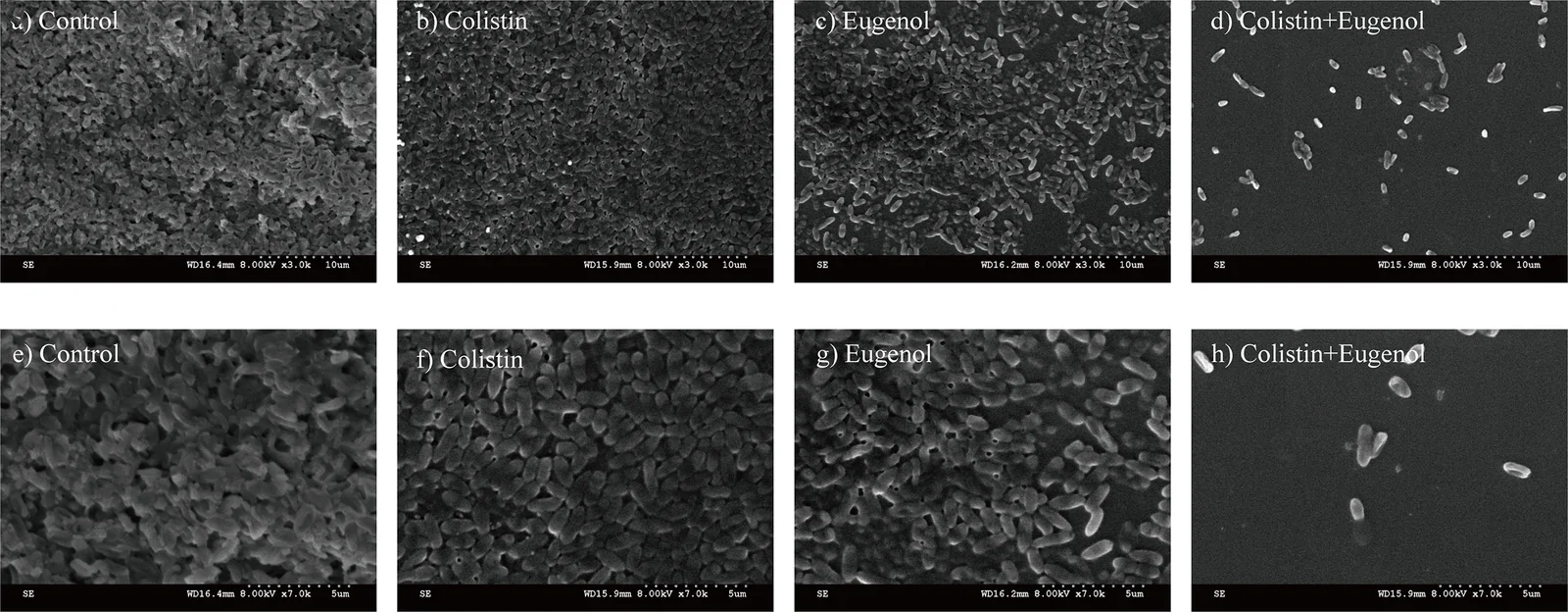
SEM image of TL2314 demonstrating the difference in biofilm and bacterial morphology between treatments. LB broth control group at 3,000× (**a**) and 7,000× (**e**); colistin (1 µg/mL) at 3,000× (**b**) and 7,000× (**f**); eugenol (125 µg/mL), at 3,000× (**c**) and 7,000× (**g**); eugenol (125 µg/mL) combined with colistin (1 µg/mL) at 3,000× (**d**) and 7,000× (**h**). COL, colistin; EG, eugenol.

Visualized images of biofilm were captured by scanning electron microscopy (SEM). Figure 4 depicts the 3,000× and 7,000× images of various treatments. Bacteria in control group completely occupy the field of vision, overlap, and crisscross to create a tight membrane structure (Fig. 4a and e). Groups treated with 1 µg/mL of colistin or 125 µg/mL of eugenol also generated integrated biofilms containing numerous microorganisms (Fig. 4b, c, f and g). However, with the combination treatment, biofilm development was significantly reduced, bacteria load was decreased, and morphology was harmed.

### *In vitro* cytotoxicity analysis

**Fig 5:**
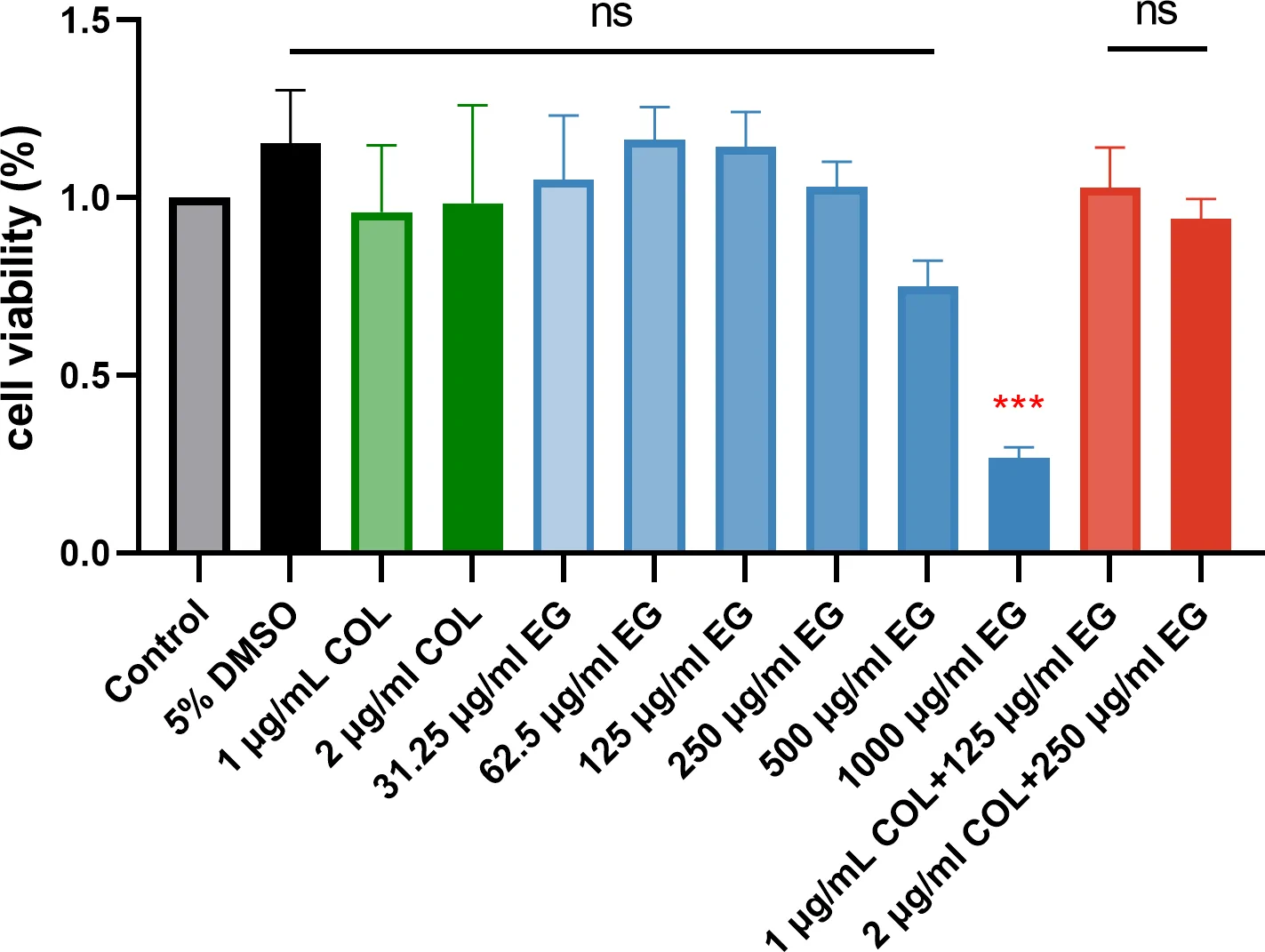
Cytotoxicity of eugenol at various doses against RAW 264.7 murine macrophage cell line. ***P < 0.001. COL, colistin; EG, eugenol; ns, nonsignificance.

We investigated the toxicity of eugenol alone and in combination with RAW 264.7 and RBCs. In Fig. 5a, up to 500 µg/mL of eugenol had no significant effect on cell viability when compared to the control and vehicle groups. This demonstrated that the concentration of two drugs in our study was safe.

### *G. mellonella* infection model

**Fig 6:**
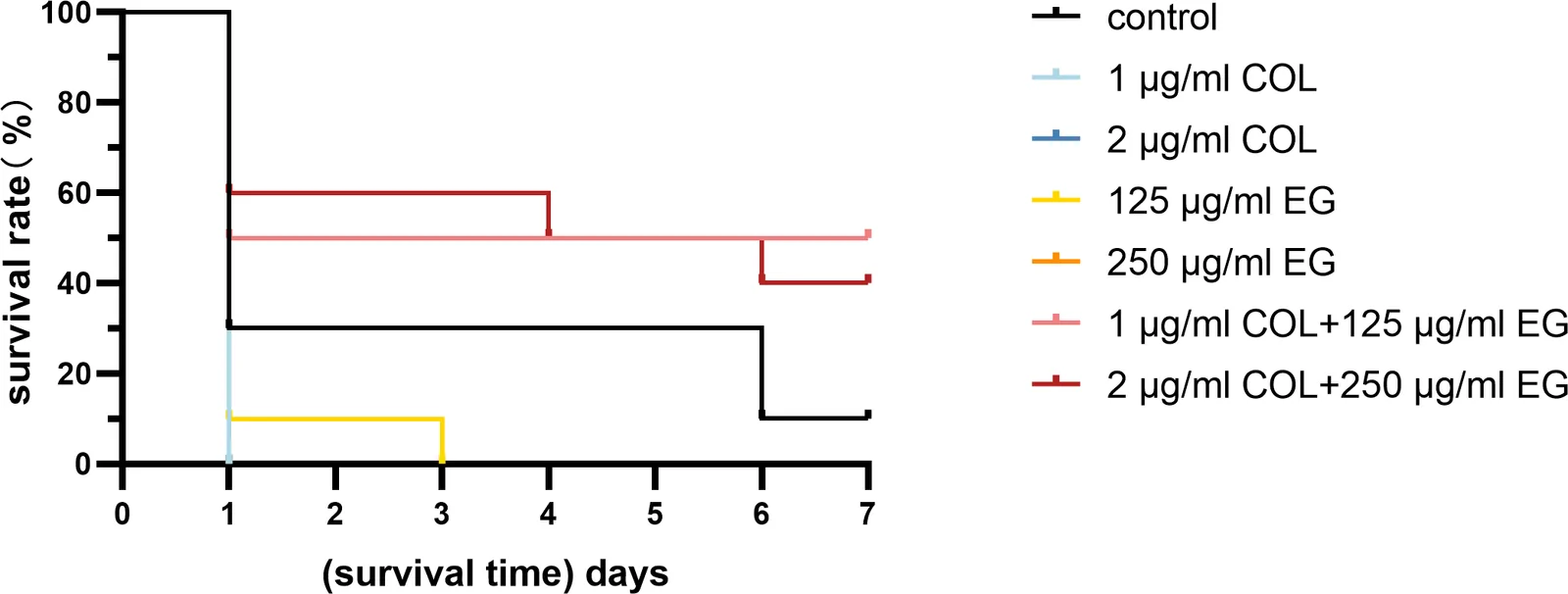
Survival rate of *G. mellonella* after various therapies. The experimental strains were and TL2917, and the survival rate of *G. mellonella* was measured after 7 days. COL, colistin; EG, eugenol.

A *G. mellonella* infection model was created to confirm the efficacy of colistin/eugenol combination against colistin-resistant bacteria. Figure 6 shows that monotherapy does not improve s survival rates and may even hasten the death of *G. mellonella* larvae. In contrast, colistin/eugenol combination treatments delayed larvae death and increased the survival rates of *G. mellonella* by 20–30%.

### Potential mechanism of synergy

**Fig 7:**
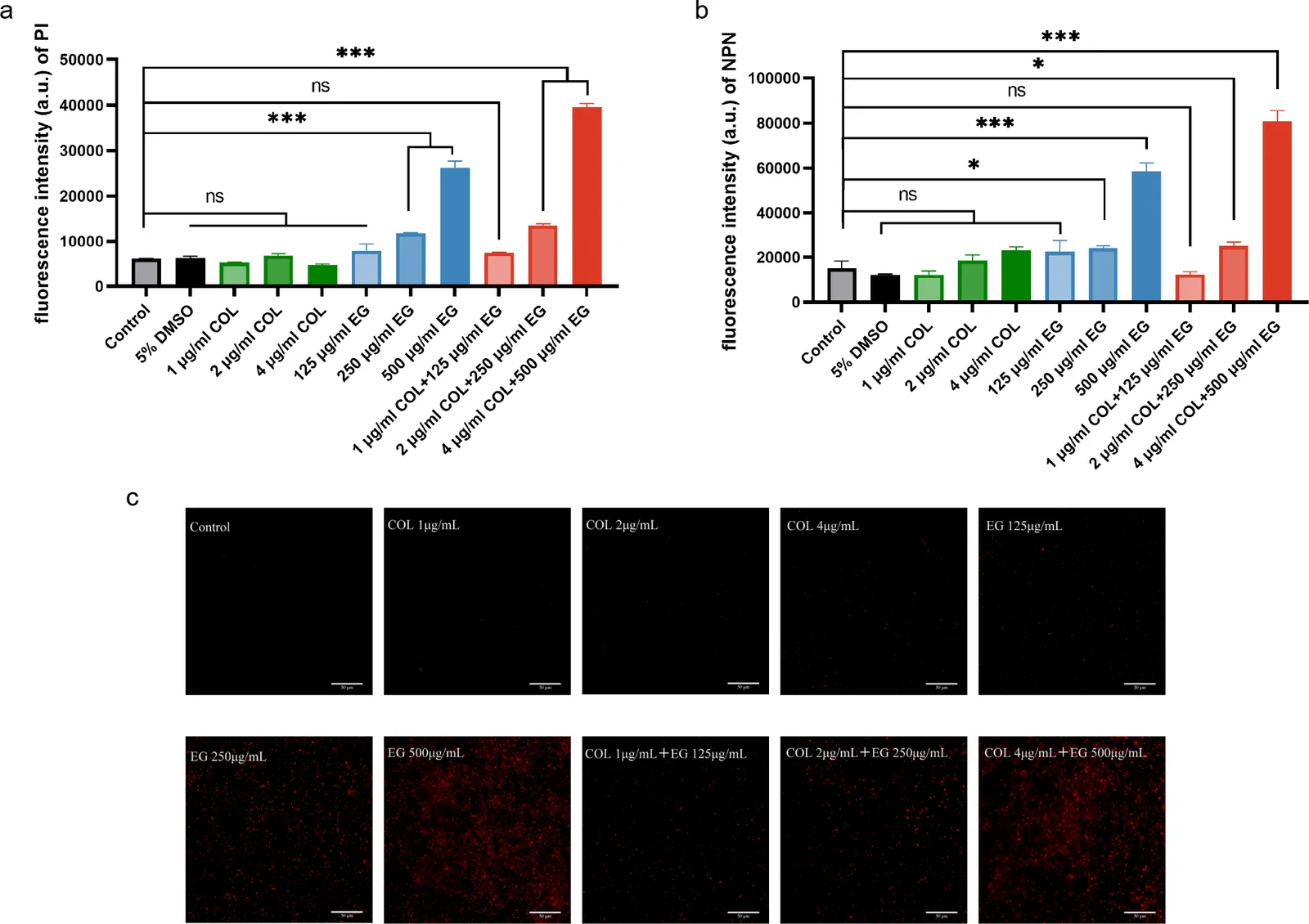
Membrane permeability assay. The fluorescence intensity (**a**) and image (**c**) of propidium iodide (PI) staining. The fluorescence intensity (**b**) of N-phenyl-1-naphthylamine (NPN) uptake. *P* < 0.05 (*), *P* < 0.01 (**), and *P* < 0.001 (***) denote significance. COL, colistin; EG, eugenol; ns, nonsignificance

**Fig 8:**
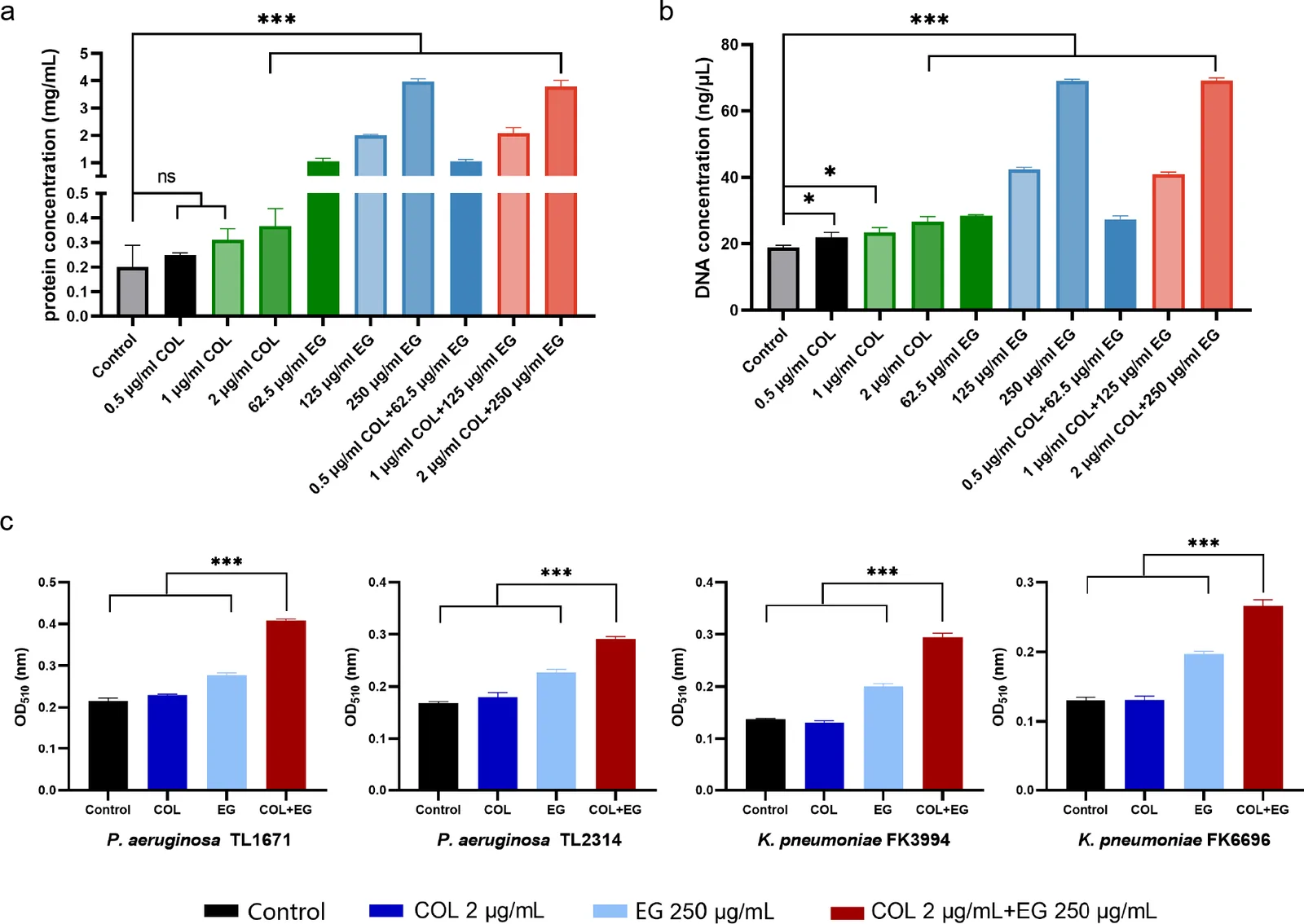
The leakage of protein (**a**), DNA (**b**), and ALPs (**c**) with different treatments, *P* < 0.05 (*), *P* < 0.01 (**), and *P* < 0.001 (***) denote significance. COL, colistin; EG, eugenol; ns, nonsignificance.

The clinical isolates TL2314 were utilized to investigate the membrane permeability using Propidium iodide (PI) staining and quantifying N-phenyl-1-naphthylamine (NPN) absorption. PI is a membrane-impermeable probe that binds to the nucleic acid of membrane-damaged bacteria, and NPN is a hydrophobic probe that can detect the permeability of the outer membrane (22). The fluorescence intensity and image of PI staining revealed a dose-dependent increase in inner membrane permeability (Fig. 7a and c), whereas the fluorescence intensity of NPN revealed a change in the outer membrane (Fig. 7b). In addition, the leakage of protein and DNA was significant increased in the presence of eugenol (Fig. 8a and b). The following alkaline phosphatases (ALPs) leakage assays also showed that the content of ALP in the bacterial supernatant increased significantly in the presence of eugenol (Fig. 8c). According to these findings, eugenol can cause significant membrane damage in bacteria, which explains its synergistic effect when used in combination with colistin.

## DISCUSSION

Bacterial resistance has evolved and expanded rapidly in recent years as a result of widespread use of many antibiotics, posing a serious danger to public health (23). Colistin remains its activity against MDR Gram-negative bacteria, it is typically used in combination with other antibiotics to treat carbapenem-resistant Gram-negative bacteria (24). Colistin with a positive charge electrostatically interacts with lipid A phosphate group of lipopolysaccharide, causing damage to the integrity of the outer membrane and spreading to the periplasm, where it disrupts the inner membrane, causing cross bonding between the inner and outer membrane, osmotic pressure imbalance, cell lysis, and death (25). However, clinical isolates of colistin-resistant strains are growing due to the reuse of colistin (26). As a result, discovering effective ways to eliminate colistin resistance is an important subject. In this study, we evaluated the efficacy of colistin combined with eugenol against colistin-resistant *P. aeruginosa* and *K. pneumoniae*.

Eugenol is one compound of natural EOs that is widely used in dentistry due to its antibacterial, anti-inflammatory, and anesthetic effects, making it a common ingredient in dental luting materials (27). Eugenol contains a hydroxyl group and unsaturated double-bond structure, which makes it susceptible to oxidation when exposed to air. Therefore, to preserve its stability, it should be stored in sealed containers and kept away from light and high temperatures (28). Clinical colistin-resistant strains are generally MDR; thus, it is critical to develop efficient strategies to prevent resistance propagation. Previous work has reported synergistic or additive antibacterial activity (FICI ranges from 0.375 to 0.625) of colistin/eugenol combination against *mcr-1* positive *E. coli*, eugenol can reduce *mcr-1* expression levels and bind to zinc atoms and serine sites of MCR-1 protein, which explains the synergy potential of eugenol from both transcription and atomic perspectives (20). In our research, we confirmed the synergistic effect and anti-biofilm effect of colistin/eugenol combination against clinical colistin-resistant *P. aeruginosa* and *K. pneumoniae in vitro* and *in vivo*. Furthermore, we analyzed the mechanism of synergy from the perspective of cell membrane destruction.

In the presence of eugenol, 12 of the 14 isolates recovered their susceptibility to colistin Table 2. Even for colistin-susceptible bacteria, eugenol can significantly potent the activity of colistin (Table S1). The dose of colistin would be reduced in combination therapy, perhaps reducing adverse effects of colistin (29). We further performed a time-kill assay to determine the dynamic antibacterial activity of colistin/eugenol against colistin-resistant *P. aeruginosa* and *K. pneumoniae*. The results demonstrated that the combination inhibits and even eliminates colistin-resistant bacteria *in vitro* (Fig. 1). In addition, the resistance to colistin of these strains is attributed to various molecular mechanisms, including the presence of the *mcr-1* gene, the mutations in the two-component systems PmrA/B and PhoP/Q, and point mutations in MgrB (30), providing important evidence for the synergistic effect of eugenol in eliminating bacterium with different colistin-resistant mechanism.

Biofilm-associated bacterial infection is a troublesome problem to manage, and is the major cause of persistent infection (31, 32). We investigated the effect of the combination on biofilm development and eradication due to eugenol’s QS inhibitory action. The combination significantly prevented the production of biofilm in seven out of eight tested isolates and could eradicate the colonized biofilm of partial isolates (three out of eight tested isolates) as compared to other treatments. SEM images revealed that the colistin/eugenol group had lower biofilm density and bacteria load.

**Fig 9:**
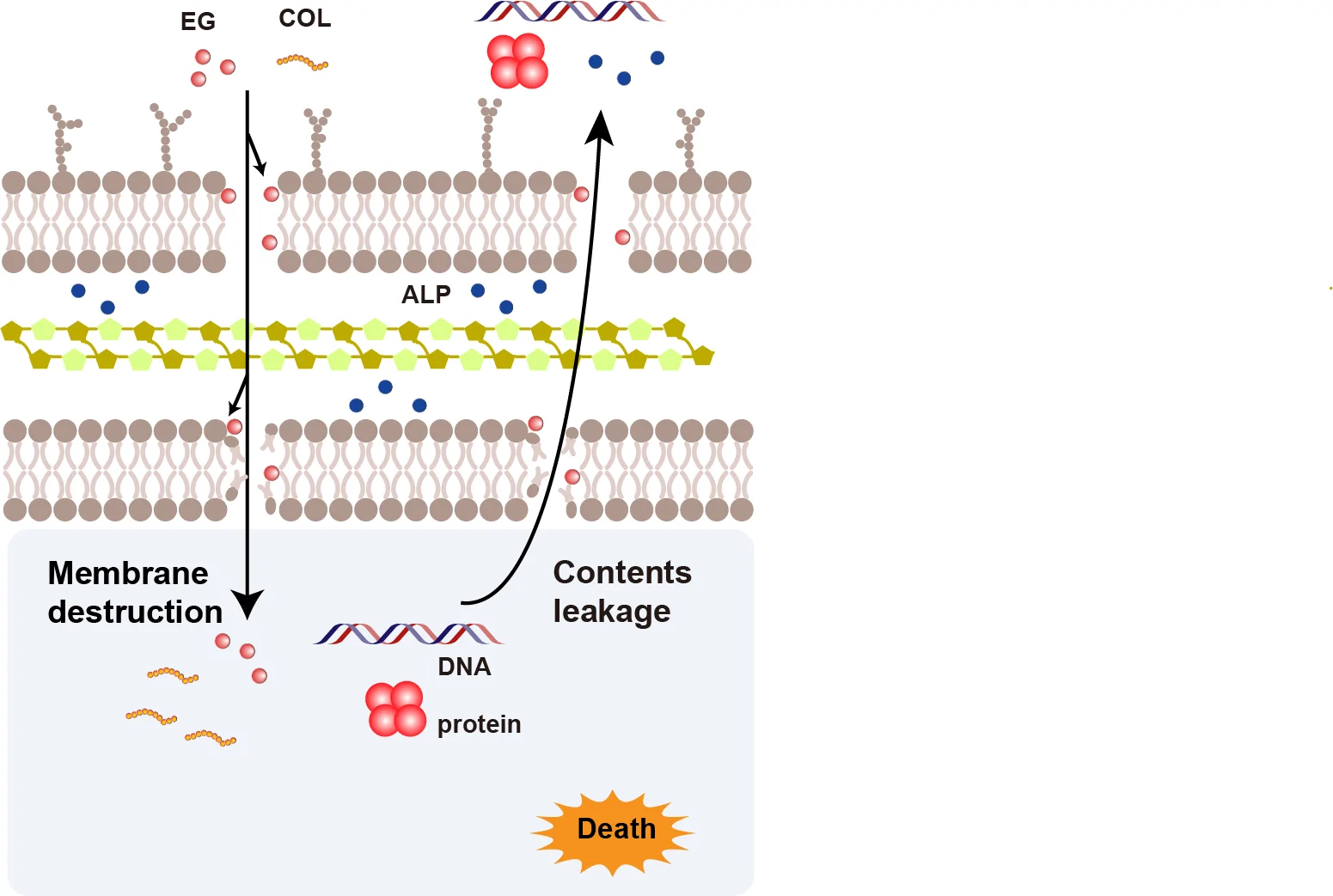
hypothesized mechanism of eugenol/colistin combination in killing colistin-resistant *P. aeruginosa* and *K. pneumoniae*.

The outer membrane of Gram-negative bacteria is a major barrier to the effectiveness of antibacterial agents. It acts as a passive barrier that prevents the penetration of drugs into the bacteria and can also actively expel drugs through efflux pumps (33, 34). Natural compounds with hydrophobic properties are able to penetrate the bacterial cell membrane and disrupt its structure, which can overcome bacterial resistance to antibiotics (35, 36). In this research, membrane-impermeable fluorescent probes (PI and NPN) were utilized to assess the integrity of membrane, revealing substantial damage to both the inner and outer membranes. In terms of intracellular substances, the presence of eugenol led to a pronounced increase in the leakage of bacterial proteins and DNA. The leakage assays of ALPs further supported the disruption of cell membrane integrity caused by eugenol, resulting in the release of bacterial contents. These findings suggest that eugenol can synergistically enhance the antibacterial effect of colistin by inducing an incomplete bacterial membrane, leading to the leakage of the bacterial contents (Fig. 9).

The safety of natural compounds is an important consideration in their usage. In our study, eugenol was not significantly toxic to RAW 264.7 at experimental concentrations. In addition, through *G. mellonella* infection model, the combined treatment delayed the death of larvae and increased the survival rates by 20–30%. These results indicate the availability of colistin/eugenol combination.

Combination therapy using antibiotics and adjuvants has been proven effective in treating MDR bacteria (37). Although natural compounds may not exhibit significant antibacterial activity on their own, they can be used as adjuvants to conventional drugs to enhance antimicrobial properties. The use of adjuvants in combination with antibiotics can overcome antibiotic resistance, prevent its spread, and reduce drug adverse effects (38). However, in the case of the eugenol/colistin combination, the high doses of eugenol used in the study pose a challenge for clinical applications. The previous studies demonstrated that intramuscular injection of 500 mg/kg eugenol resulted in an increase in the relative weight of the kidney and liver in mice, without changes in diet, activity, or appearance (39). Furthermore, pharmacokinetic analysis revealed that eugenol was primarily metabolized in the liver and excreted through the kidneys, with a rapid increase in blood concentration after oral administration (40). Therefore, further studies are needed to evaluate the safety of eugenol for clinical use. Currently, several eugenol-related nanocapsules, including nanocoated, nanoparticle, and liposome formulations, have been extensively developed to improve the biocompatibility, stability, and bioactivity of eugenol (41, 42). The development of eugenol nanopreparations will optimize its performance as an antibiotic adjuvant.

## MATERIALS AND METHODS

### Isolates, *c*ulture conditions, and reagents

A total of non-duplicate eight colistin-resistant *P. aeruginosa* and six colistin-resistant *K. pneumoniae* strains were isolated from the First Affiliated Hospital of Wenzhou Medical University. Matrix-assisted laser desorption/ionization time-of-flight mass spectrometry (MALDI-TOF/MS; bioMérieux, Lyons, France) was used to identify the isolates. ATCC 25922 and ATCC 27853 served as quality control isolates. Isolates were preserved in Luria-Bertani (LB) broth supplemented with 30% glycerol at −80°C. For further use, isolates were streaked on columbia blood agar plate (BAP) under the culture condition of 35°C. Antibiotics, including aztreonam, ceftazidime, cefepime, imipenem, ciprofloxacin, levofloxacin, gentamicin, tobramycin, avibactam, and colistin, were purchased from Wenzhou Kangtai Biotechnology Co., Ltd (Zhejiang, China). Antibiotic solvents were referred to the Clinical and Laboratory Standards Institute (CLSI 2022 M32) (43). Eugenol (MedChem Express, USA) was dissolved in 5% (vol/vol) DMSO for further usage.

### Antimicrobial susceptibility tests

The micro broth dilution method was used to assess the susceptibility of 14 colistin-resistant *P. aeruginosa* and *K. pneumoniae* clinical isolates to nine regularly used antibiotics and eugenol (43). The details are as follows, 0.5 MacFarland suspension was prepared from a single colony on BAP by sterile saline and diluted to 10^6^ CFU/mL with cationic-adjusted Mueller-Hinton broth (CAMHB; OXIOD, Britain). About 100 µL of bacterial suspension was introduced to equivalent CAMHB containing antibiotics ranging from 0.06 to 128 μg/mL and eugenol ranging from 0.25 to 1,000 μg/mL. After 16–20 h of incubation at 37°C, the MICs value was determined. According to the antibiotics breakpoints of (43) M32, to interpret susceptible, intermediate, and resistant. Each MIC test was triple-verified.

### Checkerboard assays

The synergistic effect of colistin and eugenol was investigated using checkerboard assays, as previously described but with minor modifications (44). For short, 0.125–128 μg/mL colistin was added along the horizontal axis and 15.5–1,000 μg/mL eugenol was added along the vertical axis to set several concentration combinations in 96-well plates. About 100 µL of 10^6^ CFU/mL, bacterial suspension was mixed with drug combinations to a final concentration approximately 5 × 10^5^ CFU/mL. The negative control was wells exclusively with broth. After 16–20 h of incubation at 37°C, the MICs value was determined. Each isolate was tested three times.

The FICI values were calculated (44): FIC_colistin_ = MIC_colistin_ in combination/MIC_colistin_ alone; FIC_eugenol_ = MIC_eugenol_ in combination/MIC_eugenol_ alone; FICI = FIC_colistin_ + FIC_eugenol_. Combinatorial effects were interpreted as follows: FICI ≤ 0.5 indicated synergism; 0.5 < FICI ≤ 1 indicated additive effect; 1 < FICI ≤ 2 indicated irrelevant effect; FICI > 2 indicated antagonistic effect.

### Time-kill assays

Colistin-resistant *P. aeruginosa* (TL1671, TL2314, TL3008, and TL3086) and *K. Pneumoniae* (FK1342, FK3994, FK6663, and FK6696) isolates were randomly tested for growth kinetic curves of colistin-resistant isolates under various treatments, as previously published (45). Briefly, colistin at a final concentration of 1–2 μg/mL and eugenol at a final concentration of 125–250 μg/mL, either alone or in combination, were introduced into 20 mL LB broth containing 10^6^ CFU/mL bacteria. The concentrations of these two drugs were determined using checkerboard assay with FICI ≤ 0.5. About 16 µg/mL of CAZ/AVI and blank groups were used as positive control and growth control, respectively. Tubes were shaken at 180 rpm and incubated at 37°C. At 0, 2, 4, 6, 12, and 24 h, CFU were counted on LB plate. Synergistic activity was determined as a ≥2 log_10_ CFU/mL reduction in the combination when compared to other groups.

### Biofilm formation inhibition assays

Eight isolates of colistin-resistant *P. aeruginosa* (TL1671, TL2314, TL3008, and TL3086) and *K. pneumoniae* (FK1342, FK3994, FK6663, and FK6696) were tested in biofilm formation inhibition assays as previously described but with modification (46). A single colony was shaken in 3 mL LB broth overnight, and the bacterial suspension was adjusted to 0.5 MacFarland and before being diluted 100-fold with LB broth. About 100 µL suspension was mixed with an equal LB broth containing colistin, eugenol, or their corresponding combination. The concentration was determined using the checkerboard assay findings for *P. aeruginosa*, colistin 1 µg/mL, eugenol 250 µg/mL, and *K. pneumoniae*, colistin 1 µg/mL, eugenol 125 µg/mL group without drugs served as growth control. The 96-well plates were then incubated at 37°C for 24 h. After incubation, planktonic bacteria were rinsed away two times with 1× phosphate-buffered saline (PBS). The plate was naturally dried, and the biofilm was stained with 0.1% crystal violet for 15 min before being rinsed three times with 1× PBS, and dissolved in 200 µL ethanol-acetone (95:5 vol/vol). The OD_595nm_ was measured on a microplate reader. The experiment was repeated three times.

### Colonized biofilm eradication assays

Crystal violet staining was used to investigate the effect of colistin/eugenol combination on mature biofilm in colistin-resistant *P. aeruginosa* (TL1671, TL2314, TL3008, and TL3086) and *K. pneumoniae* (FK1342, FK3994, FK6663, and FK6696) (47). Briefly, a single colony was introduced in 3 mL of LB broth for shaking overnight, and the bacterial suspension was adjusted to 0.5 MacFarland before being diluted 100-fold with LB broth. About 200 µL of bacterial suspension was added to sterile blank 96-well plates, and the plates were incubated at 37°C for 24 h. The planktonic cells were separated and washed. The prepared colistin and eugenol solvents were introduced into the wells and incubated at 37℃ for 24 h. The dyeing procedure is the same as previously, including crystal violet staining and OD_595nm_ measurement. The experiment was carried out three times.

### Scanning electron microscopy

SEM was used to demonstrate the intuitive alteration of colistin/eugenol combination on biofilm of *P. aeruginosa* TL2314. Briefly, 2 mL LB broth of bacterial suspension (5 × 10^6^ CFU/mL) containing1 μg/mL of colistin, 125 µg/mL of eugenol or their combination were added to six-well plates, followed by sterile glass slides (9 mm × 9 mm) put into each well. Well without drugs served as growth control. After incubating at 37°C for 24 h, slides were removed, washed three times with PBS, and bacteria of adhesion were fixed with 2.5% (vol/vol) glutaraldehyde (Solarbio, Beijing) at 4°C for 4 h. Then solution was then diluted and dehydrated in stages with graded ethanol (30%, 50%, 70%, 90%, and 100% vol/vol) for 15 min each (48). The sample was air-dried at room temperature before being coated with platinum, and then observed by SEM (S-3000N, Japan).

### *G. mellonella* infection model

*G. mellonella* survival rates were used to assess the efficiency of the colistin/eugenol combination *in vivo*, as previously described with minor modifications (49). FK6696 was adjusted to 0.5 MacFarland and further diluted to 10^5^ CFU/mL. A microinjector was used to inject bacterial suspension (10 µL) into the back left proleg of 250–350 mg larvae (10 larvae per group). Monotherapy with 1 and 2 µg/mL of colistin, 125 and 250 µg/mL of eugenol, or combined therapy with 1 µg/mL of colistin and 125 µg/mL of eugenol, and 2 µg/mL of colistin with 250 µg/mL of eugenol were injected (10 µL) after 2 h of infection. *G. mellonella* survival rates were measured at 24, 48, 72, 96, 120, 144, and 168 h. Larvae that darkened bodies or had no reaction to repeated stimuli were assumed to be dead. The Kaplan-Meier analysis and the log-rank test were adopted to analyze *G. mellonella* mortality rates.

### Cytotoxicity assays

For the cytotoxicity test, RAW 264.7 cells were employed, and adherent cells were cultured in Dulbecco’s modified Eagle’s medium (DMEM) supplemented with 10% heat-inactivated fetal bovine serum in a CO_2_ incubator at 37°C. Each well of the 96-well microplates received 100 µL of cell suspension containing 10^5^ cells. After incubated for 24 h, wells were treated with 10 µL serial concentration of colistin (1 and 2 µg/mL), eugenol (31, 62, 125, 250, 500, and 1,000 µg/mL) or their combinations (1 µg/mL of colistin and 125 µg/mL of eugenol, or 2 µg/mL of colistin and 250 µg/mL of eugenol). Cells treated with 5% DMSO or left untreated served as vehicle and negative control. After 12–18 h of incubation, 10 µL of CCK-8 (Solarbio) was added.

2-(2-Methoxy-4-nitrophenyl)−3-(4-nitrophenyl)−5-(2,4-disulfophenyl)−2H-tetrazolium Sodium Salt (WST-8, key component of CCK-8 kit) can be REDOX to water-soluble yellow Formazan by NAD+, the more viable cells, the more Formazan were produced. OD_450nm_ was measured prior to incubating for 1 h in a CO_2_ incubator. Cell viability (%) was calculated as follows: *A*_1_−*A*_0_/*A*_2_−*A*_0_ × 100%, *A*_1_, *A*_2_, and *A*_0_ indicate distinct treatment groups, untreated groups, and wells exclusively with DMEM and CCK-8, respectively.

### Propidium iodide staining and N-phenyl-1-naphthylamine uptake assays

Inner and outer membrane permeabilities were measured using propidium iodide staining and N-phenyl-1-naphthylamine fluorochromes, as previously described and with modifications (22). Logarithmic phase *P. aeruginosa* TL2314 cells were treated for 2 h with a single drug (1, 2, and 4 µg/mL of colistin and 125, 250, and 500 µg/mL of eugenol) or in combination, and then incubated with PI (50 µg/mL) or NPN (50 µg/mL) at 37°C for 30 min. Fluorescence intensity was measured using a multifunctional microplate reader (BioTek) at excitation of 535 nm and emission of 615 nm for PI and excitation of 350 nm, and emission of 420 nm for NPN. Images of PI staining were captured using a Fluorescence Microscope (Nikon, Japan). Experiments were carried out three times.

### Protein and leakage assays

To clarify the effect of colistin/eugenol combination on the intracellular component, we detected the leakage of bacterial proteins and DNA. The suspension of *P. aeruginosa* TL2314 was adjusted to an optical density of approximately OD_600nm_ ≈ 0.6 and subsequently resuspended in PBS three times. The bacterial suspension was then incubated with colistin alone, eugenol alone and the colistin/eugenol combination for 6 h. Following centrifugation at 12,000 × *g* for 10 min, the concentration of protein and DNA in the supernatant was determined using a nanodrop spectrophotometer. Experiments were carried out three times.

### Alkaline phosphatase leakage assays

To study the leakage of periplasm contents, ALPs leakage assays were carried out, according to the previously described (50). Log-phase of *P. aeruginosa* (TL1671 and TL2314) and *K. pneumoniae* (FK3994 and FK6696) were introduced into LB broth containing 250 µg/mL of eugenol, 2 µg/mL of colistin, and 250 µg/mL of eugenol + 2 µg/mL of colistin for 37°C 6 h shaking incubation. Then, suspensions were centrifuged at 5,000 rpm for 5 min. The ALPs of supernatants were detected with a commercial kit (Solarbio). ALP catalyzed the formation of free phenol from substrate, which then reacted with Potassium Ferricyanide and 4-Aminoantipyrine to form Quinone Derivatives; the absorbance was measured at 510 nm. Experiments were performed in triplicate.

### Quantitative Real-Time PCR

The expression of *mcr-1* gene (F 5′-TGCTCCAAAATGCCCTACAGACC-3′, R 5′-TGCCCCAAGTCGGATAATCCAC-3′) was assessed using quantitative real-time PCR analysis. The internal reference gene used for normalization was 16S rRNA (F 5′-TGTCGTCAGCTCGTGTTGTG-3′, R 5′-ATCCCCACCTTCCTCCAGTT-3′). Total RNA was extracted from the *K. pneumoniae* strains FK6663 cultured in LB broth containing colistin, eugenol, or colistin + eugenol for 16–18 h using a commercial RNA extraction kit (Tiangen Biotech, Beijing, China), following the manufacturer’s instructions. Gene expression was quantified using the 2^−ΔΔCt^ method, and this experiment was conducted in triplicate.

### Statistical analysis

Data were presented as mean ± standard deviation. One-way analysis of variance was used to assess the statistical significance of differences between control and experimental groups. *P* value < 0.05 indicated statistical significance. GraphPad Prism 8.0 was used for the statistical analysis.

### Conclusion

This is the first report to study the synergistic activity of colistin and eugenol against *P. aeruginosa* and *K. pneumoniae in vitro* and *in vivo*. Eugenol acts as a membrane-damaging adjuvant, increasing colistin sensitivity significantly.

## SUPPLEMENTAL MATERIAL

Figure S1 spectrum.03666-22-s0001.tif The mcr-1 gene expression.

Table S1 spectrum.03666-22-s0002.docx Synergy against carbapenem-resistant strains.

Tables S2 and S3 spectrum.03666-22-s0003.docx MIC of carbapenem-resistant strains.

ASM does not own the copyrights to Supplemental Material that may be linked to, or accessed through, an article. The authors have granted ASM a non-exclusive, world-wide license to publish the Supplemental Material files. Please contact the corresponding author directly for reuse.
