# Supplementary figures and images for: Eugenol works synergistically with colistin against colistin-resistant Pseudomonas aeruginosa and Klebsiella pneumoniae isolates by enhancing membrane permeability
Source: Microbiol Spectr. 2023 Sep 14;11(5):e03666-22. doi: 10.1128/spectrum.03666-22 (PMC10581171; doi:10.1128/spectrum.03666-22)

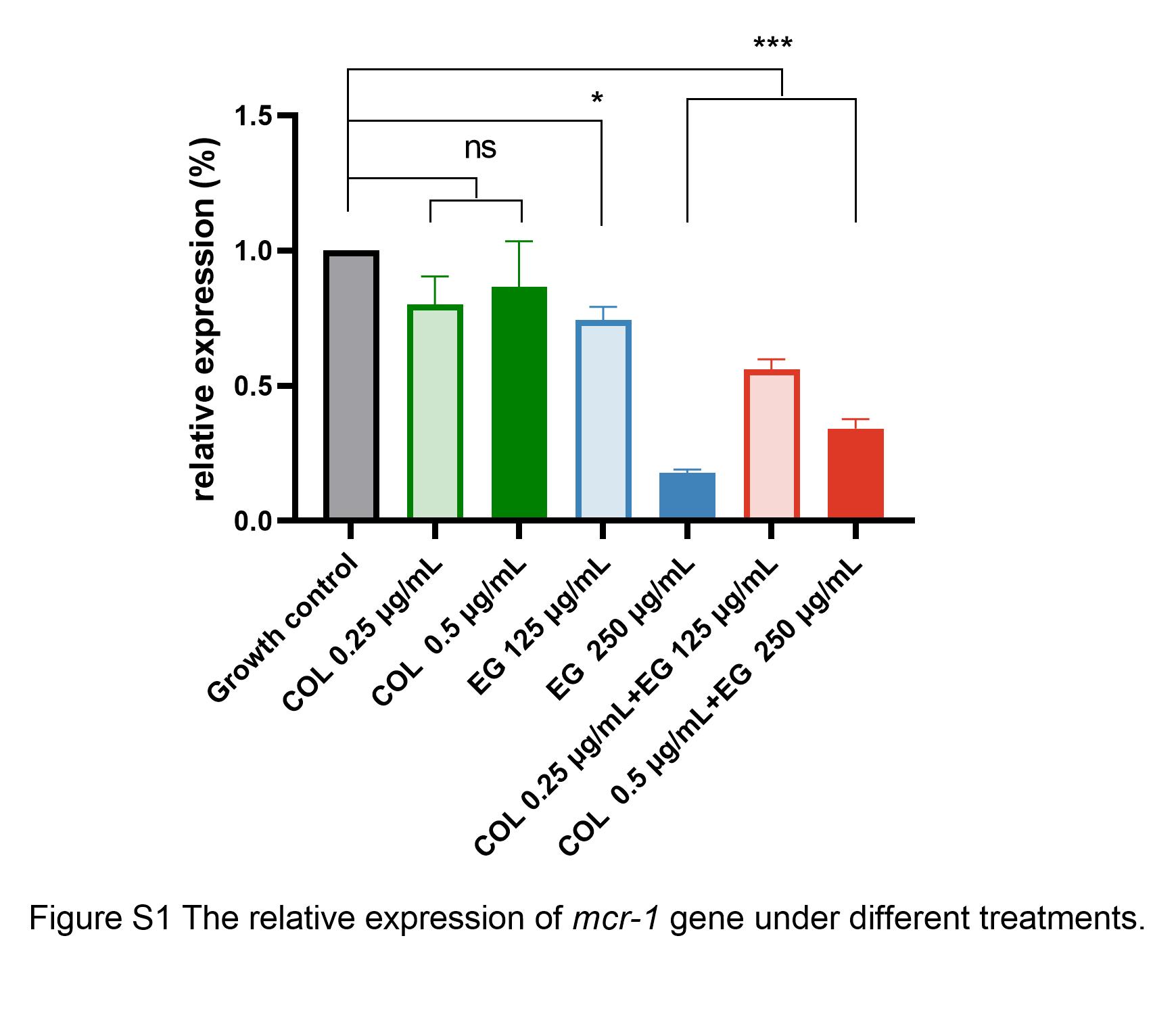

Supplement: Figure S1 — The mcr-1 gene expression. [file spectrum.03666-22-s0001.tif]
