# Supplementary material for: Eugenol works synergistically with colistin against colistin-resistant Pseudomonas aeruginosa and Klebsiella pneumoniae isolates by enhancing membrane permeability
Source: Microbiol Spectr. 2023 Sep 14;11(5):e03666-22. doi: 10.1128/spectrum.03666-22 (PMC10581171; doi:10.1128/spectrum.03666-22)
Supplement: Table S1 — Synergy against carbapenem-resistant strains. [file spectrum.03666-22-s0002.docx]

**Table S1** The MICs and FICIs value for colistin/eugenol combination against colistin-susceptible and carbapenem-resistant strains.

| Isolates | MIC  (μg/ml) | | FIC  (μg/ml) | | FICI | Interaction |
| --- | --- | --- | --- | --- | --- | --- |
|  | Colistin | Eugenol | Colistin | Eugenol |  |  |
| TL2912-OMV | 0.5 | >1000 | 0.12 | 31.25 | **< 0.281** | **Synergy** |
| TL3144-OMV | 0.5 | >1000 | 0.25 | 31.25 | < 0.531 | Addition |
| TL3570 | 0.5 | >1000 | 0.12 | 125 | **< 0.375** | **Synergy** |
| TL3652 | 0.5 | >1000 | 0.12 | 125 | **< 0.375** | **Synergy** |
| TL3683 | 0.5 | >1000 | 0.12 | 125 | **< 0.375** | **Synergy** |
| TL3773 | 1 | >1000 | 0.25 | 125 | **< 0.375** | **Synergy** |
| TL3777 | 0.5 | >1000 | 0.25 | 31.25 | < 0.531 | Addition |
| TL3783 | 0.5 | 1000 | 0.12 | 250 | **0.5** | **Synergy** |
| TL3788 | 0.5 | >1000 | 0.25 | 62.5 | < 0.563 | Addition |
| TL3593 | 0.5 | >1000 | 0.25 | 62.5 | < 0.563 | Addition |
| FK6709 | 0.016 | 1000 | 0.008 | 62.5 | 0.563 | Addition |
| FK7942 | 0.06 | >1000 | 0.03 | 125 | < 0.625 | Addition |
| FK8052 | 0.06 | >1000 | 0.016 | 62.5 | **< 0.313** | **Synergy** |
| FK8113 | 0.06 | >1000 | 0.03 | 62.5 | < 0.563 | Addition |
| FK8160 | 0.06 | >1000 | 0.016 | 125 | **< 0.375** | **Synergy** |
| FK8271 | 0.06 | >1000 | 0.016 | 125 | **< 0.375** | **Synergy** |
| FK8355 | 0.06 | 1000 | 0.03 | 62.5 | 0.563 | Addition |
| FK8410 | 0.25 | 1000 | 0.03 | 31.25 | **0.156** | **Synergy** |
| FK8699 | 0.03 | 1000 | 0.016 | 500 | 1 | Addition |
| FK8839 | 0.12 | >1000 | 0.03 | 250 | **< 0.5** | **Synergy** |
