# Supplementary material for: Eugenol works synergistically with colistin against colistin-resistant Pseudomonas aeruginosa and Klebsiella pneumoniae isolates by enhancing membrane permeability
Source: Microbiol Spectr. 2023 Sep 14;11(5):e03666-22. doi: 10.1128/spectrum.03666-22 (PMC10581171; doi:10.1128/spectrum.03666-22)
Supplement: Tables S2 and S3 — MIC of carbapenem-resistant strains. [file spectrum.03666-22-s0003.docx]

**Table S2** The MIC values (µg/mL) or Zone Diameters (mm) of colistin-susceptible and carbapenem-resistant *P. aeruginosa* strains.

| strains | CAZ | FEP | IPM | MEM | GEN | CIP | TZP | SCF | COL |
| --- | --- | --- | --- | --- | --- | --- | --- | --- | --- |
| TL2912-OMV | 8 | 8 | 32^R^ | 8^R^ | ≤1 | 2 | 64 | ND | 0.5 |
| TL3144-OMV | 16 | ≤1 | 16^R^ | 4^R^ | ≤1 | ≤0.25 | 64 | ND | 0.5 |
| TL3570 | ≥64^R^ | ≥64^R^ | ≥16^R^ | 6^R^ | 2 | ≥4^R^ | ≥128^R^ | 7^R^ | 0.5 |
| TL3652 | ≥64^R^ | 32^R^ | ≥16^R^ | 6^R^ | 2 | 2 | ≥128^R^ | 12^R^ | 0.5 |
| TL3683 | ≥64^R^ | 16 | ≥16^R^ | 12^R^ | 2 | 2 | 64 | 16 | 0.5 |
| TL3773 | ≥64^R^ | ≥64^R^ | ≥16^R^ | ≥16^R^ | 8 | ≥4^R^ | ≥128^R^ | 6^R^ | 1 |
| TL3777 | 16 | 16 | ≥16^R^ | 6^R^ | ≥16^R^ | ≥4^R^ | ≥128^R^ | 16 | 0.5 |
| TL3783 | ≥64^R^ | ≥64^R^ | ≥16^R^ | 12^R^ | 4 | 2 | ≥128^R^ | 16 | 0.5 |
| TL3788 | 16 | 16 | 8^R^ | 6^R^ | ≤1 | 0.5 | 64 | 16 | 0.5 |
| TL3593 | ≥64^R^ | 16 | ≥16^R^ | 9^R^ | ≤1 | 0.5 | 64 | 17 | 0.5 |

CAZ, Ceftazidime; FEP, Cefepime; IPM, Imipenem; MEM, Meropenem; GEN, Gentamicin; CIP, Ciprofloxacin ; TZP, Piperacillin-tazobactam; SCF, Cefperazone-Sulbactam; COL, Colistin; ND, not detection. MIC values (µg/mL) or Zone Diameters (mm) are shown in black and blue font, respectively.

**Table S3** The MIC values (µg/mL) or Zone Diameters (mm) of colistin-susceptible and carbapenem-resistant *K. pneumoniae* strains.

| strains | ATM | CAZ | FEP | ETP | IPM | GEN | CIP | TZP | SCF | SXT | COL |
| --- | --- | --- | --- | --- | --- | --- | --- | --- | --- | --- | --- |
| FK6709 | ≥64^R^ | ≥64^R^ | ≥64^R^ | ≥8^R^ | ≥16^R^ | ≤1 | ≥4^R^ | ≥128^R^ | 6^R^ | ≥320^R^ | 0.016 |
| FK7942 | ≥64^R^ | ≥64^R^ | ≥64^R^ | ≥8^R^ | ≥16^R^ | ≥16^R^ | ≥4^R^ | ≥128^R^ | 8^R^ | ≥320^R^ | 0.06 |
| FK8052 | 16^R^ | 15^R^ | 19 | 6^R^ | 14^R^ | ≤1 | ≤0.25 | ≥128^R^ | 16 | ≤20 | 0.06 |
| FK8113 | ≤1 | 8 | ≤1 | 15^R^ | 23 | ≤1 | ≥4^R^ | 8 | 20 | 160^R^ | 0.06 |
| FK8160 | ≥64^R^ | ≥64^R^ | ≥64^R^ | ≥8^R^ | ≥16^R^ | ≥16^R^ | ≥4^R^ | ≥128^R^ | 6^R^ | ≤20 | 0.06 |
| FK8271 | ≥64^R^ | 16^R^ | 17^R^ | 15^R^ | 15^R^ | ≤1 | ≤0.25 | ≥128^R^ | 16 | ≤20 | 0.06 |
| FK8355 | ≥64^R^ | ≥64^R^ | ≥64^R^ | ≥8^R^ | ≥16^R^ | ≤1 | ≥4^R^ | ≥128^R^ | 6^R^ | ≥320^R^ | 0.06 |
| FK8410 | ≥64^R^ | ≥64^R^ | ≥64^R^ | 6^R^ | 12^R^ | ≤1 | 0.5 | 11^R^ | 8^R^ | ≥320^R^ | 0.25 |
| FK8699 | ≥64^R^ | ≥64^R^ | ≥64^R^ | ≥8^R^ | ≥16^R^ | ≥16^R^ | ≥4^R^ | ≥128^R^ | 6^R^ | ≤20 | 0.03 |
| FK8839 | ≥64^R^ | ≥64^R^ | ≥64^R^ | ≥8^R^ | 8^R^ | ≥16^R^ | ≥4^R^ | ≥128^R^ | 9^R^ | ≥320^R^ | 0.12 |

ATM, aztreonam; CAZ, Ceftazidime; FEP, Cefepime; ETP, Ertapenem; IPM, Imipenem; GEN, Gentamicin; CIP, Ciprofloxacin ;TZP, Piperacillin-tazobactam; SCF, Cefperazone-Sulbactam; SXT, Trimethoprim-sulfamethoxazole; COL, Colistin. MIC values (µg/mL) or Zone Diameters (mm) are shown in black and blue font, respectively.
